# (2*E*)-1-(2-Bromo­phen­yl)-3-(4-meth­oxy­phen­yl)prop-2-en-1-one

**DOI:** 10.1107/S160053681002218X

**Published:** 2010-06-16

**Authors:** Jerry P. Jasinski, Ray J. Butcher, K. Veena, B. Narayana, H. S. Yathirajan

**Affiliations:** a Department of Chemistry, Keene State College, 229 Main Street, Keene, NH 03435-2001, USA; b Department of Chemistry, Howard University, 525 College Street NW, Washington DC 20059, USA; c Department of Studies in Chemistry, Mangalore University, Mangalagangotri, 574 199, India; d Department of Studies in Chemistry, University of Mysore, Manasagangotri, Mysore 570 006, India

## Abstract

In the title compound, C_16_H_13_BrO_2_, two benzene rings form a dihedral angle of 44.3 (9)°. In the crystal, weak inter­molecular C—H⋯O hydrogen bonds link the mol­ecules into chains propagating in [010]. The crystal packing also exhibits short Br⋯Br contacts of 3.4787 (8) Å. A comparison of the DFT-optimized gas-phase mol­ecular geometry with that in the crystal structure revealed only small differences.

## Related literature

For the radical quenching properties of included phenol groups, see: Dhar (1981 ▶). For related structures, see: Arai *et al.* (1994 ▶); Li *et al.* (1992 ▶); Patil *et al.* (2007 ▶); Shettigar *et al.* (2006 ▶). For standard bond lengths, see Allen *et al.* (1987 ▶). For density functional theory, see: Schmidt & Polik (2007 ▶); Hehre *et al.* (1986 ▶).
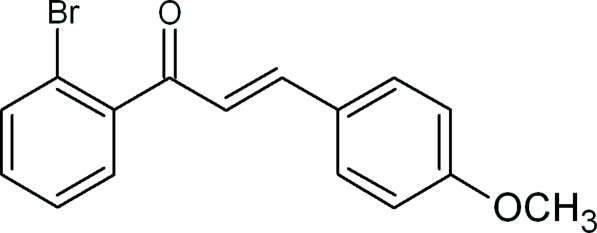


## Experimental

### 

#### Crystal data

C_16_H_13_BrO_2_
*M* *_r_* = 317.17
Monoclinic, 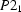

*a* = 12.7300 (8) Å
*b* = 4.0061 (3) Å
*c* = 13.0035 (6) Å
β = 100.671 (5)°
*V* = 651.68 (7) Å^3^
*Z* = 2
Cu *K*α radiation
μ = 4.25 mm^−1^
*T* = 110 K
0.48 × 0.41 × 0.28 mm

#### Data collection

Oxford Diffraction Xcalibur diffractometer with Ruby (Gemini Cu) detector
Absorption correction: multi-scan (*CrysAlis RED*; Oxford Diffraction, 2007 ▶) *T*_min_ = 0.423, *T*_max_ = 1.000
2117 measured reflections
1641 independent reflections
1621 reflections with *I* > 2σ(*I*)
*R*_int_ = 0.018

#### Refinement

*R*[*F* ^2^ > 2σ(*F* ^2^)] = 0.034
*wR*(*F* ^2^) = 0.091
*S* = 1.07
1641 reflections
173 parameters
1 restraint
H-atom parameters constrained
Δρ_max_ = 0.71 e Å^−3^
Δρ_min_ = −0.65 e Å^−3^
Absolute structure: Flack (1983 ▶), 133 Friedel pairs
Flack parameter: 0.00 (3)

### 

Data collection: *CrysAlis PRO* (Oxford Diffraction, 2007 ▶); cell refinement: *CrysAlis PRO*; data reduction: *CrysAlis PRO*; program(s) used to solve structure: *SHELXS97* (Sheldrick, 2008 ▶); program(s) used to refine structure: *SHELXL97* (Sheldrick, 2008 ▶); molecular graphics: *SHELXTL* (Sheldrick, 2008 ▶); software used to prepare material for publication: *SHELXTL*.

## Supplementary Material

Crystal structure: contains datablocks I. DOI: 10.1107/S160053681002218X/cv2728sup1.cif

Structure factors: contains datablocks I. DOI: 10.1107/S160053681002218X/cv2728Isup2.hkl

Additional supplementary materials:  crystallographic information; 3D view; checkCIF report

## Figures and Tables

**Table 1:** Hydrogen-bond geometry (Å, °)

*D*—H⋯*A*	*D*—H	H⋯*A*	*D*⋯*A*	*D*—H⋯*A*
C12—H12*A*⋯O1^i^	0.95	2.59	3.541 (5)	174

Symmetry code: (i) 
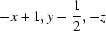
.

**Fig. 1.**
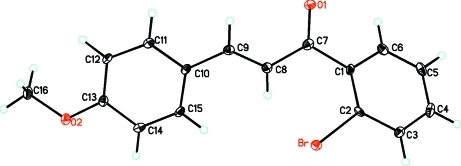
Molecular structure of (I) showing the atom labeling scheme and 50% probability displacement ellipsoids.

**Fig. 2.**
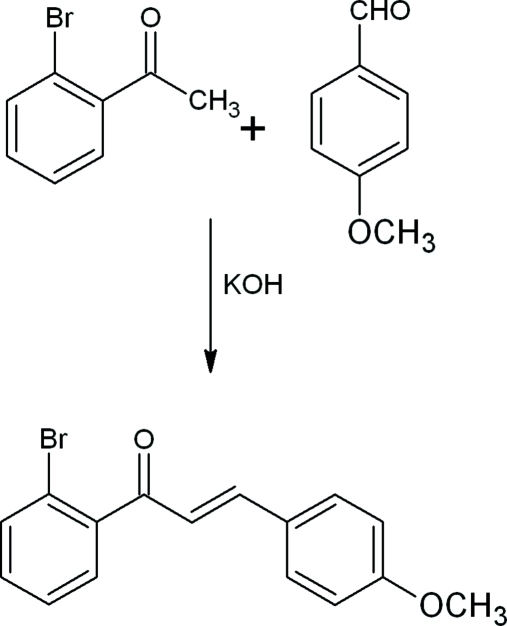
Scheme for the synthesis of (I).

## supplementary crystallographic information

### Comment

Chalcones, or 1,3-diaryl-2-propen-1-ones, belong to the flavonoid family. Chemically they consist of open-chain flavonoids in which the two aromatic rings are joined by a three-carbon α,β-unsaturated carbonyl system. A vast number of naturally occurring chalcones are polyhydroxylated in the aryl rings. The radical quenching properties of the phenolic groups present in many chalcones have raised interest in using the compounds or chalcone rich plant extracts as drugs or food preservatives (Dhar, 1981). The crystal structures of some closely related chalcones, *viz*., 1-(4-bromophenyl)-3-(3-methoxy-phenyl)prop-2-en-1-one (Patil *et al.*, 2007); 4-bromo-4'-methoxy-chalcone (Li *et al.*, 1992), 4-bromo-4'-methoxychalcone (Arai *et al.*, 1994) and 1-(4-bromophenyl)-3-(4-methoxyphenyl)prop-2-en-1-one (Shettigar *et al.*, 2006) have been reported. Hence in continuation with the synthesis and crystal structure determination and also owing to the importance of these flavonoid analogs, this new bromo chalcone, (I), C_16_H_13_BrO_2_, is synthesized and its crystal structure is reported.

The title compound, (I), C_16_H_13_BrO_2_, is a chalcone with 4-methoxyphenyl and 2-bromophenyl rings bonded at opposite rings of a propene group (Fig. 1). The dihedral angle between mean planes of the *para*-methoxy and *ortho*-bromo substituted benzene rings is 44.3 (9)°. The angles between the mean plane of the prop-2-ene-1-one group and the mean planes of the 4-meyhoxyphenyl and 2-bromophenyl rings are 6.3 (1)° and 44.6(36°, respectively. Bond distances and angles are in normal ranges (Allen *et al.*, 1987). While no classical hydrogen bonds are present, a weak intermolecular C12—H12A···O1 interaction (Table 1) is observed which contributes to the stability of crystal packing.

A density functional theory (DFT) geometry optimization molecular orbital calculation (Schmidt & Polik, 2007) was performed on (I) with the B3LYP 6–31-G(*d*) basis set (Hehre *et al.*, 1986). The dihedral angle between mean planes of the *para*-methoxy and *ortho*-bromo substituted benzene rings becomes 45.98°, an increase of 1.59°. The angles between the mean plane of the prop-2-ene-1-one group and the mean planes of the 4-meyhoxyphenyl and 2-bromophenyl rings become 3.65° and 42.40°, changes of -2.65° and +0.74°, respectively. These observations suggest that the weak intermolecular C12—H12A···O1 interaction produces a small effect on crystal stability.

### Experimental

A 50% KOH solution was added to a mixture of 2-bromo acetophenone (0.01 mol, 1.99 g) and 4-methoxy benzaldehyde (0.01 mol, 1.36 g) in 25 ml of ethanol (Fig. 2). The mixture was stirred for an hour at room temperature and the precipitate was collected by filtration and purified by recrystallization from ethanol. The single-crystal was grown from ethyl acetate by slow evaporation method and yield of the compound was 50% (m.p.336–338 K). Analytical data, composition (%): found (calculated): C: 60.52 (60.59%); H: 4.10 (4.13%).

### Refinement

The H atoms were placed in geometrically idealized positions and constrained to ride on their parent atoms, with C–H distances = 0.95–0.98Å and with *U*_iso_(H) = 1.17–1.47 *U*_eq_(C).

### Crystal data

**Table:**

C_16_H_13_BrO_2_	*F*(000) = 320
*M**_r_* = 317.17	*D*_x_ = 1.616 Mg m^−^^3^
Monoclinic, *P*2_1_	Cu *K*α radiation, λ = 1.54184 Å
Hall symbol: P 2yb	Cell parameters from 1981 reflections
*a* = 12.7300 (8) Å	θ = 4.5–74.1°
*b* = 4.0061 (3) Å	µ = 4.25 mm^−^^1^
*c* = 13.0035 (6) Å	*T* = 110 K
β = 100.671 (5)°	Chunk, colourless
*V* = 651.68 (7) Å^3^	0.48 × 0.41 × 0.28 mm
*Z* = 2	

### Data collection

**Table:**

Oxford Diffraction Xcalibur diffractometer with Ruby (Gemini Cu) detector	1641 independent reflections
Radiation source: Enhance (Cu) X-ray Source	1621 reflections with *I* > 2σ(*I*)
graphite	*R*_int_ = 0.018
Detector resolution: 10.5081 pixels mm^-1^	θ_max_ = 74.1°, θ_min_ = 4.5°
ω scans	*h* = −9→15
Absorption correction: multi-scan (*CrysAlis RED*; Oxford Diffraction, 2007)	*k* = −2→4
*T*_min_ = 0.423, *T*_max_ = 1.000	*l* = −15→16
2117 measured reflections	

### Refinement

**Table:**

Refinement on *F*^2^	Secondary atom site location: difference Fourier map
Least-squares matrix: full	Hydrogen site location: inferred from neighbouring sites
*R*[*F*^2^ > 2σ(*F*^2^)] = 0.034	H-atom parameters constrained
*wR*(*F*^2^) = 0.091	*w* = 1/[σ^2^(*F*_o_^2^) + (0.0718*P*)^2^ + 0.5775*P*] where *P* = (*F*_o_^2^ + 2*F*_c_^2^)/3
*S* = 1.07	(Δ/σ)_max_ = 0.001
1641 reflections	Δρ_max_ = 0.71 e Å^−^^3^
173 parameters	Δρ_min_ = −0.65 e Å^−^^3^
1 restraint	Absolute structure: Flack (1983), 133 Friedel pairs
Primary atom site location: structure-invariant direct methods	Flack parameter: 0.00 (3)

### Special details

**Table:**

Experimental. IR data (KBr) ν cm^-1^: 2998 cm^-1^, 2937 cm^-1^, 2839 cm^-1^ (C—H al.str), 3058 cm^-1^ (C—H ar. str) 1646 cm^-1^ (C=O), 1580 cm^-1^ (C=C); 1245 cm^-1^ (C—O—C).
Geometry. All e.s.d.'s (except the e.s.d. in the dihedral angle between two l.s. planes) are estimated using the full covariance matrix. The cell e.s.d.'s are taken into account individually in the estimation of e.s.d.'s in distances, angles and torsion angles; correlations between e.s.d.'s in cell parameters are only used when they are defined by crystal symmetry. An approximate (isotropic) treatment of cell e.s.d.'s is used for estimating e.s.d.'s involving l.s. planes.
Refinement. Refinement of *F*^2^ against ALL reflections. The weighted *R*-factor *wR* and goodness of fit *S* are based on *F*^2^, conventional *R*-factors *R* are based on *F*, with *F* set to zero for negative *F*^2^. The threshold expression of *F*^2^ > σ(*F*^2^) is used only for calculating *R*-factors(gt) *etc*. and is not relevant to the choice of reflections for refinement. *R*-factors based on *F*^2^ are statistically about twice as large as those based on *F*, and *R*- factors based on ALL data will be even larger.

### Fractional atomic coordinates and isotropic or equivalent isotropic displacement parameters (Å^2^)

**Table:**

	*x*	*y*	*z*	*U*_iso_*/*U*_eq_	
Br	0.38764 (3)	0.18817 (18)	0.46303 (2)	0.01538 (15)	
O1	0.2178 (2)	0.2427 (10)	0.1406 (2)	0.0211 (8)	
O2	0.8860 (2)	0.2977 (9)	0.1659 (2)	0.0209 (7)	
C1	0.2190 (3)	0.4727 (13)	0.3077 (3)	0.0149 (9)	
C2	0.2577 (3)	0.4233 (12)	0.4142 (3)	0.0119 (8)	
C3	0.2022 (3)	0.5312 (12)	0.4896 (3)	0.0166 (9)	
H3A	0.2299	0.4917	0.5616	0.020*	
C4	0.1060 (3)	0.6971 (19)	0.4599 (3)	0.0223 (8)	
H4A	0.0679	0.7740	0.5116	0.027*	
C5	0.0651 (3)	0.7513 (12)	0.3545 (3)	0.0211 (11)	
H5A	−0.0008	0.8660	0.3342	0.025*	
C6	0.1203 (3)	0.6385 (14)	0.2797 (3)	0.0187 (10)	
H6A	0.0913	0.6732	0.2078	0.022*	
C7	0.2730 (3)	0.3590 (12)	0.2195 (3)	0.0173 (9)	
C8	0.3891 (3)	0.4058 (13)	0.2303 (3)	0.0160 (9)	
H8A	0.4255	0.5526	0.2824	0.019*	
C9	0.4441 (3)	0.2431 (12)	0.1673 (3)	0.0161 (10)	
H9A	0.4037	0.1037	0.1154	0.019*	
C10	0.5595 (3)	0.2564 (10)	0.1697 (3)	0.0145 (11)	
C11	0.5999 (3)	0.1064 (11)	0.0878 (3)	0.0164 (10)	
H11A	0.5519	−0.0064	0.0344	0.020*	
C12	0.7083 (3)	0.1171 (11)	0.0822 (3)	0.0155 (10)	
H12A	0.7334	0.0191	0.0248	0.019*	
C13	0.7787 (3)	0.2733 (11)	0.1619 (3)	0.0156 (10)	
C14	0.7411 (4)	0.4183 (13)	0.2470 (3)	0.0186 (9)	
H14A	0.7897	0.5209	0.3022	0.022*	
C15	0.6327 (3)	0.4102 (13)	0.2495 (3)	0.0175 (9)	
H15A	0.6075	0.5107	0.3065	0.021*	
C16	0.9272 (3)	0.1512 (18)	0.0810 (3)	0.0223 (11)	
H16A	0.9121	−0.0888	0.0783	0.033*	
H16B	1.0046	0.1870	0.0916	0.033*	
H16C	0.8930	0.2553	0.0151	0.033*	

### Atomic displacement parameters (Å^2^)

**Table:**

	*U*^11^	*U*^22^	*U*^33^	*U*^12^	*U*^13^	*U*^23^
Br	0.0159 (2)	0.0153 (2)	0.0148 (2)	−0.0005 (2)	0.00224 (13)	0.00195 (19)
O1	0.0204 (13)	0.028 (2)	0.0151 (11)	−0.0044 (15)	0.0031 (10)	−0.0034 (14)
O2	0.0169 (14)	0.027 (2)	0.0207 (14)	−0.0012 (13)	0.0070 (12)	−0.0027 (13)
C1	0.0154 (18)	0.016 (2)	0.0154 (17)	−0.0033 (18)	0.0081 (15)	−0.0001 (17)
C2	0.0083 (16)	0.008 (2)	0.0179 (17)	0.0027 (16)	−0.0026 (13)	0.0032 (17)
C3	0.021 (2)	0.014 (2)	0.0160 (18)	−0.0060 (18)	0.0067 (15)	−0.0023 (16)
C4	0.0242 (18)	0.018 (2)	0.0292 (19)	−0.004 (3)	0.0173 (15)	−0.006 (3)
C5	0.0136 (17)	0.016 (3)	0.034 (2)	0.0014 (18)	0.0047 (16)	0.0002 (19)
C6	0.0165 (17)	0.016 (3)	0.0228 (17)	−0.003 (2)	0.0023 (14)	0.0024 (19)
C7	0.023 (2)	0.015 (2)	0.0152 (18)	−0.0028 (19)	0.0061 (16)	0.0040 (17)
C8	0.0176 (19)	0.015 (3)	0.0155 (17)	−0.0006 (18)	0.0036 (14)	0.0011 (17)
C9	0.0189 (17)	0.016 (3)	0.0131 (15)	−0.0011 (18)	0.0025 (13)	0.0027 (18)
C10	0.0197 (18)	0.014 (3)	0.0113 (15)	0.0016 (17)	0.0058 (14)	0.0018 (15)
C11	0.0208 (19)	0.015 (3)	0.0135 (16)	0.0001 (17)	0.0037 (14)	0.0000 (15)
C12	0.0200 (18)	0.015 (3)	0.0124 (15)	0.0016 (17)	0.0062 (14)	−0.0020 (15)
C13	0.0179 (18)	0.014 (3)	0.0159 (17)	0.0019 (16)	0.0068 (14)	0.0034 (16)
C14	0.024 (2)	0.019 (2)	0.0122 (16)	−0.001 (2)	0.0024 (15)	−0.0014 (18)
C15	0.022 (2)	0.020 (3)	0.0116 (16)	0.002 (2)	0.0071 (15)	0.0001 (18)
C16	0.0181 (17)	0.025 (3)	0.0261 (18)	0.000 (2)	0.0101 (14)	−0.004 (2)

### Geometric parameters (Å, °)

**Table:**

Br—C2	1.907 (4)	C8—H8A	0.9500
O1—C7	1.224 (5)	C9—C10	1.464 (5)
O2—C13	1.361 (5)	C9—H9A	0.9500
O2—C16	1.433 (5)	C10—C11	1.401 (6)
C1—C2	1.396 (5)	C10—C15	1.403 (6)
C1—C6	1.408 (6)	C11—C12	1.396 (6)
C1—C7	1.512 (5)	C11—H11A	0.9500
C2—C3	1.380 (6)	C12—C13	1.387 (6)
C3—C4	1.384 (7)	C12—H12A	0.9500
C3—H3A	0.9500	C13—C14	1.410 (6)
C4—C5	1.390 (6)	C14—C15	1.387 (6)
C4—H4A	0.9500	C14—H14A	0.9500
C5—C6	1.379 (6)	C15—H15A	0.9500
C5—H5A	0.9500	C16—H16A	0.9800
C6—H6A	0.9500	C16—H16B	0.9800
C7—C8	1.471 (6)	C16—H16C	0.9800
C8—C9	1.341 (6)		
			
C13—O2—C16	116.7 (3)	C8—C9—H9A	116.4
C2—C1—C6	117.3 (4)	C10—C9—H9A	116.4
C2—C1—C7	125.7 (4)	C11—C10—C15	117.6 (4)
C6—C1—C7	117.1 (4)	C11—C10—C9	118.5 (4)
C3—C2—C1	121.9 (4)	C15—C10—C9	123.9 (4)
C3—C2—Br	116.4 (3)	C12—C11—C10	122.2 (4)
C1—C2—Br	121.7 (3)	C12—C11—H11A	118.9
C2—C3—C4	119.7 (4)	C10—C11—H11A	118.9
C2—C3—H3A	120.2	C13—C12—C11	118.9 (4)
C4—C3—H3A	120.2	C13—C12—H12A	120.6
C3—C4—C5	120.1 (4)	C11—C12—H12A	120.6
C3—C4—H4A	120.0	O2—C13—C12	124.5 (4)
C5—C4—H4A	120.0	O2—C13—C14	115.1 (4)
C6—C5—C4	119.9 (4)	C12—C13—C14	120.4 (4)
C6—C5—H5A	120.1	C15—C14—C13	119.5 (4)
C4—C5—H5A	120.1	C15—C14—H14A	120.2
C5—C6—C1	121.3 (4)	C13—C14—H14A	120.2
C5—C6—H6A	119.4	C14—C15—C10	121.3 (4)
C1—C6—H6A	119.4	C14—C15—H15A	119.3
O1—C7—C8	122.6 (4)	C10—C15—H15A	119.3
O1—C7—C1	118.7 (4)	O2—C16—H16A	109.5
C8—C7—C1	118.6 (4)	O2—C16—H16B	109.5
C9—C8—C7	120.5 (4)	H16A—C16—H16B	109.5
C9—C8—H8A	119.7	O2—C16—H16C	109.5
C7—C8—H8A	119.7	H16A—C16—H16C	109.5
C8—C9—C10	127.2 (4)	H16B—C16—H16C	109.5
			
C6—C1—C2—C3	0.1 (7)	C1—C7—C8—C9	164.4 (4)
C7—C1—C2—C3	−179.0 (4)	C7—C8—C9—C10	−178.4 (4)
C6—C1—C2—Br	177.7 (4)	C8—C9—C10—C11	−170.9 (4)
C7—C1—C2—Br	−1.3 (7)	C8—C9—C10—C15	9.4 (7)
C1—C2—C3—C4	−0.8 (7)	C15—C10—C11—C12	−2.4 (6)
Br—C2—C3—C4	−178.6 (4)	C9—C10—C11—C12	177.9 (4)
C2—C3—C4—C5	0.7 (8)	C10—C11—C12—C13	1.9 (6)
C3—C4—C5—C6	0.2 (9)	C16—O2—C13—C12	0.2 (7)
C4—C5—C6—C1	−1.1 (8)	C16—O2—C13—C14	179.6 (5)
C2—C1—C6—C5	0.9 (7)	C11—C12—C13—O2	179.4 (4)
C7—C1—C6—C5	180.0 (4)	C11—C12—C13—C14	0.0 (6)
C2—C1—C7—O1	139.4 (5)	O2—C13—C14—C15	179.2 (4)
C6—C1—C7—O1	−39.6 (7)	C12—C13—C14—C15	−1.4 (7)
C2—C1—C7—C8	−43.2 (7)	C13—C14—C15—C10	0.8 (7)
C6—C1—C7—C8	137.8 (5)	C11—C10—C15—C14	1.0 (7)
O1—C7—C8—C9	−18.2 (7)	C9—C10—C15—C14	−179.3 (4)

### Hydrogen-bond geometry (Å, °)

**Table:**

*D*—H···*A*	*D*—H	H···*A*	*D*···*A*	*D*—H···*A*
C12—H12A···O1^i^	0.95	2.59	3.541 (5)	174

Symmetry codes: (i) −*x*+1, *y*−1/2, −*z*.
